# Repeatability of derived parameters from histograms following non-Gaussian diffusion modelling of diffusion-weighted imaging in a paediatric oncological cohort

**DOI:** 10.1007/s00330-016-4318-2

**Published:** 2016-03-22

**Authors:** Neil P. Jerome, Keiko Miyazaki, David J. Collins, Matthew R. Orton, James A. d’Arcy, Toni Wallace, Lucas Moreno, Andrew D. J. Pearson, Lynley V. Marshall, Fernando Carceller, Martin O. Leach, Stergios Zacharoulis, Dow-Mu Koh

**Affiliations:** 1 Division of Radiotherapy & Imaging, The Institute of Cancer Research, Cancer Research UK Cancer Imaging Centre, 123 Old Brompton Road, London, SW7 3RP UK; 2 Department of Radiology, Royal Marsden NHS Foundation Trust, Sutton, Surrey SM2 5PT UK; 3 Paediatric Drug Development Team, Division of Cancer Therapeutics and Clinical Studies, The Institute of Cancer Research, 123 Old Brompton Road, London, SW7 3RP UK; 4 Hospital Niño Jesus, Av Menendez Pelayo 65, Madrid, Spain; 5 Paediatric Drug Development Unit, Children and Young People’s Unit, Royal Marsden NHS Foundation Trust, Sutton, Surrey SM2 5PT UK

**Keywords:** Diffusion magnetic resonance imaging, Reproducibility of results, Functional magnetic resonance imaging, Paediatrics, Medical oncology

## Abstract

**Objectives:**

To examine repeatability of parameters derived from non-Gaussian diffusion models in data acquired in children with solid tumours.

**Methods:**

Paediatric patients (<16 years, *n* = 17) were scanned twice, 24 h apart, using DWI (6 *b*-values, 0–1000 mm^−2^ s) at 1.5 T in a prospective study. Tumour ROIs were drawn (3 slices) and all data fitted using IVIM, stretched exponential, and kurtosis models; percentage coefficients of variation (CV) calculated for each parameter at all ROI histogram centiles, including the medians.

**Results:**

The values for ADC, *D*, DDC_α_, α, and DDC_K_ gave CV < 10 % down to the 5th centile, with sharp CV increases below 5th and above 95th centile. *K*, *f*, and D* showed increased CV (>30 %) over the histogram. ADC, *D*, DDC_α_, and DDC_K_ were strongly correlated (ρ > 0.9), DDC_α_ and α were not correlated (ρ = 0.083).

**Conclusion:**

Perfusion- and kurtosis-related parameters displayed larger, more variable CV across the histogram, indicating observed clinical changes outside of *D*/DDC in these models should be interpreted with caution. Centiles below 5th for all parameters show high CV and are unreliable as diffusion metrics. The stretched exponential model behaved well for both DDC_α_ and α, making it a strong candidate for modelling multiple-*b*-value diffusion imaging data.

***Key Points*:**

*• ADC has good repeatability as low 5th centile of the histogram distribution.*

• *High CV was observed for all parameters at extremes of histogram.*

• *Parameters from the stretched exponential model showed low coefficients of variation.*

• *The median ADC, D, DDC*_*α*_ *, and DDC*_*K*_ *are highly correlated and repeatable.*

• *Perfusion/kurtosis parameters showed high CV variations across their histogram distributions.*

## Introduction

Diffusion-weighted imaging (DWI) is a functional magnetic resonance imaging technique that is widely used in adult clinical trials of novel anticancer therapeutics that may have cytostatic rather than cytotoxic effect [1–5]. There is less experience of implementing DWI in paediatric oncology, where introduction of new targeted anticancer therapeutics is a priority [6–8]. Similar to drug development in adults, functional imaging, including DWI, may have a significant role in providing pharmacodynamic proof-of-target inhibition, or predictive biomarkers to identify patients most likely to benefit from a specific therapy.

DWI displays contrast arising from water protons that are in motion, following the application of magnetic field gradients. This contrast informs on tissue cellularity, tortuosity of the extracellular space, and integrity of cellular membranes. Malignant tumours are characterised by increased cellularity compared to their native tissues, and the impeded water diffusion results in a lower apparent diffusion coefficient (ADC) value. DWI has potential for oncological disease characterisation [9], with ADC having been reported as a potential marker for response in high-grade paediatric brain tumours [10], but there are still very few reports of functional imaging studies in children with extracranial tumours [11]. Studies that have investigated the repeatability of functional imaging-derived parameters in adults [3, 12] do not necessarily reflect the added challenges involved when scanning children.

Clinical studies commonly report summary statistics for ADC within a region-of-interest (ROI), such as the mean or median, but alternate properties of the ADC histogram may give more insight into tumour heterogeneity and be of clinical interest [5, 13–15]. Lower ADC regions within a tumour, including the lowest centiles of the ADC histogram, may represent the highest cellularity and more aggressive disease. Beyond the simple monoexponential ADC model, more complex diffusion models may be applied to characterise observed non-monoexponential signal attenuation in tissues, potentially providing additional information relating to tumour perfusion, or empirical parameters that capture the non-Gaussian character of the water diffusion.

A recent study [16] demonstrated that in paediatric tumours, the challenges of imaging children did not adversely affect the feasibility or repeatability of median ADC measurements; it is unknown whether such observations hold true for more complex diffusion models. Further, there is interest in moving from simple summary statistics (i.e. median values) to evaluating the lowest parameter values that may reflect regions within the tumour that are the most cellular and may show differential response [17]. With increased interest in such models and the use of summary statistics beyond the mean and median, it is critical that derived diffusion metrics be assessed for repeatability in the context of parameter histograms.

The aim of this study is to evaluate the repeatability of diffusion parameters derived from non-Gaussian diffusion models in children with solid tumours, and to examine the repeatability of each diffusion model parameter at different centiles of the parameter histogram across the tumour ROI. Specifically, the bi-exponential intravoxel incoherent motion (IVIM) model [18], the stretched exponential model [19], and the kurtosis model [20] will be applied to clinically acquired multiple-*b*-value DWI data; since optimal model choice may be dependent on pathology, and while practical limitations prevent collection of a suitable range of *b* values for all DWI models, the common physiological interpretations and suitability judgements of these different models will be explicitly avoided, with parameters from repeat baseline observations being examined solely for repeatability.

## Materials and methods

### Patient population

The institutional review board (IRB) approved this prospective study, which included written consent for participation obtained from each child’s parent or legal guardian, and assent from each child, prior to inclusion. Study inclusion criteria included (a) patients under 16 years, (b) confirmed diagnosis of a solid tumour, (c) a measurable target lesion diameter of at least 2 cm, and (d) MRI included as part of their standard care. Exclusion criteria were (a) patients requiring general anaesthesia for imaging, (b) patients with impairment of renal function, (c) patients with previous allergy to contrast or any contraindications to MR imaging (this study also included DCE-MRI, and a return visit solely for repeat DWI data was not considered ethical), (d) patients with lung metastases only, and (e) patients with disease at locations likely to result in significant artefacts in diffusion imaging (motion or pulsatile artefacts, e.g. in lung or around the mediastinum). Scanning was performed pre-treatment. This study focussed on diffusion model parameter repeatability and behaviour in histogram analysis, and explicitly does not interpret the parameter values themselves, allowing a broad range of pathologies to be eligible for inclusion in the study and thus collection of a sufficient cohort. During the period January 2010 until August 2013, 17 patients with confirmed solid tumours were recruited. Monoexponential analysis of DWI images from this patient cohort has been previously reported by Miyazaki et al. [16], but was confined to the ROI median of the monoexponential diffusion model fitting (results reproduced here for reference only), and analysis of concurrently acquired DCE-MRI data not reported here.

### Diffusion MR imaging

All imaging was performed on a 1.5 T MAGNETOM Avanto MR system (Siemens Healthcare, Erlangen, Germany) using a phased-array head coil (intracranial tumours) or a phased-array body coil (extracranial tumours). DWI was performed during the routine MRI scan, and repeated after 24 h with no intervention. A free-breathing DWI protocol using a multi-slice, single-shot echo-planar imaging (EPI) sequence was used with the following parameters: TE 75 ms; TR 3500 ms; matrix 128×128 (interpolated to 256 × 256); 24 contiguous 5-mm slices; generalised autocalibrating partially parallel acquisition (GRAPPA) acceleration factor 2; spectral adiabatic inversion-recovery (SPAIR) fat suppression, and three signal averages. Diffusion *b* values of 0, 50, 100, 300, 600, and 1000 mm^−2^ s were applied in three orthogonal directions (50 mm^−2^ s being the minimum increment at the time of protocol design), and isotropic trace images calculated. The intracranial field of view was 220 × 220 mm^2^ transverse, the extracranial field of view was 300 × 300 mm^2^ coronal. The imaging volume was centred through the target lesion, and the total time for the DWI protocol was 6 min.

### Image analysis

For each patient, ROIs were manually drawn around the tumour using all available imaging, and excluding necrotic areas, by an expert radiologist (D.M.K., more than 10 years of experience) for three central slices (where possible) in each initial data set, and matching slices of the repeat data set. Image analysis was performed offline using proprietary software (ADEPT, The Institute of Cancer Research, UK). Diffusion model fitting was performed on a voxel-by-voxel basis using a Markov chain Monte Carlo approach as a robust least-squares estimator, returning the following parameters from the following models: (i) the IVIM, Eq. 1, giving the slow diffusion component *D*, the fast pseudo-diffusion component D*, the pseudo-diffusion fraction *f*, and the compound parameter fD* [21]; (ii) the stretched exponential model, Eq. 2, giving the distributed diffusion coefficient DDC_α_ and the stretching exponent α; (iii) the kurtosis model, Eq. 3, giving the diffusion coefficient DDC_K_ and the kurtosis parameter *K*. Reproduced here with permission for comparison are the results from repeatability of monoexponential fitting for *b* values of at least 100 mm^−2^ s, to give apparent diffusion coefficient ADC_100_ (Eq. 4), as reported by Miyazaki et al. [16].

1 $$ {S}_b={S}_0.\left[f. \exp \left(-b.{D}^{*}\right)+\left(1-f\right). \exp \left(-b.D\right)\right] $$

2 $$ {S}_b={S}_0. \exp \left(-{\left(b.DD{C}_{\alpha}\right)}^{\alpha}\right) $$

3 $$ {S}_b={S}_0. \exp \left(-b.DD{C}_K+\frac{1}{6}.{K}^2.{b}^2\right) $$

4 $$ {S}_b={S}_0. \exp \left(-b.ADC\right) $$

In all equations, *S*_*b*_ is the signal intensity for a given *b* value, *S*_0_ is the signal at *b* = 0 mm^−2^ s, and *b* is the applied *b* value (mm^−2^ s).

### Statistical analysis

For each patient and parameter in each diffusion model, the voxel-by-voxel results from within the tumour ROI from the three slices were combined, and the percentiles (0th to 100th) calculated in each case. Median values for each diffusion parameter were compared between the two visits (paired *t* test; statistical significance in this study defined at *p* < 0.05). The repeatability of all diffusion parameters at each percentile was found by calculating the repeated measures coefficient of variation across the cohort, expressed as a percentage (see Eq. 5), derived from σ^2^, the variance of the difference of the log-transformed measurement values [22]. Lastly, the correlation between all DWI parameters was examined using the average of the median values from the repeat measurements.

5 $$ CV=100\%\times \sqrt{ \exp \left(\frac{\sigma^2}{2}\right)-1} $$

## Results

Average patient age was 11 years (median; range 6–15 years); details of the patient characteristics and their primary tumour classification are given in Table 1. Patients were co-operative and able to tolerate the free-breathing DWI protocol. For one patient a repeat scan was logistically inconvenient, and the DWI data in one patient suffered a technical failure, leaving repeatability values of the DWI parameters derived from 15 pairs of measurements (seven intracranial, eight extracranial). The median diffusion parameters are given in Table 2 for all the models considered; the values across the cohort (given as mean ± s.d.) show a large variation, which is unsurprising given the varied pathologies included. There was no statistically significant difference in the parameters in the repeat measures (*p* > 0.1 in all cases).

**Table 1:** Patient cohort details

	Sex/age (years)	Pathology
Intracranial tumour		
1	F/8	Anaplastic astrocytoma
2	M/7	Primitive neuroectodermal tumour
3	M/10	Glioblastoma multiforme
4	M/11	High grade glioma
5	F/9	Glioblastoma multiforme
6	M/8	Glioblastoma multiforme
7	M/15	Anaplastic astrocytoma
8	M/11	Astrocytoma
Extracranial tumour		
9	M/7	Ganglioneuroblastoma
10	M/13	Spindle cell sarcoma
11	F/12	Ganglioneuroblastoma
12	M/14	Rhabdomyosarcoma
13	M/15	Sacral myxopapillary ependymoma
14	M/12	Intra-abdominal primitive neuroectodermal tumour
15	M/6	Neuroblastoma
16	M/13	Rhabdomyosarcoma
17	M/14	Neuroendocrine tumour

**Table 2:** Mean ± s.d. of tumour ROI median values for estimated DWI parameters across the cohort. High variation in these values is expected to have a contribution from the variety of pathologies included within the repeatability study

	Intracranial		Extracranial		Full cohort	
	Visit 1	Visit 2	Visit 1	Visit 2	Visit 1	Visit 2
ADC_100_ (10^−5^ mm^2^ s^−1^)	175.8 ± 45.8	174.7 ± 47.2	104.2 ± 21.1	106.4 ± 26.1	142.4 ± 51.0	142.8 ± 51.5
*f* (%)	11.6 ± 6.9	8.6 ± 6.5	7.9 ± 3.5	7.5 ± 2.2	9.9 ± 5.8	8.1 ± 4.9
*D* (10^−5^ mm^2^ s^−1^)	166.5 ± 45.7	167.3 ± 47.2	99.0 ± 23.6	100.7 ± 23.4	135.0 ± 50.0	136.2 ± 50.3
D* (10^−2^ mm^2^ s^−1^)	1.5 ± 1.1	1.5 ± 0.9	1.1 ± 0.6	0.9 ± 0.3	1.3 ± 0.9	1.2 ± 0.7
fD* (10^−4^ mm^2^ s^−1^)	23.7 ± 22.8	20.8 ± 22.1	8.1 ± 4.6	7.9 ± 3.5	16.4 ± 18.3	14.8 ± 17.1
DDCα (10^−5^ mm^2^ s^−1^)	191.6 ± 49.1	185.9 ± 43.8	105.9 ± 25.2	109.0 ± 28.9	151.6 ± 58.6	150.0 ± 53.8
α	0.9 ± 0.1	0.9 ± 0.1	0.9 ± 0.1	0.9 ± 0.0	0.9 ± 0.1	0.9 ± 0.1
DDC_K_ (10^−5^ mm^2^ s^−1^)	225.8 ± 59.1	216.6 ± 46.5	123.3 ± 24.6	119.8 ± 17.1	178.0 ± 69.4	171.4 ± 60.9
Kurtosis (mm^4^ s^−2^)	1.1 ± 0.5	1.1 ± 0.6	1.4 ± 0.8	1.5 ± 0.6	1.3 ± 0.7	1.3 ± 0.7

The coefficients of variation (CV) and 95 % confidence interval limits for the medians of all the derived diffusion imaging parameters are summarised in Table 3, with reference values from monoexponential fitting [16]. Of all the parameters considered from the different models, the most reproducible diffusion parameter for the full cohort was IVIM-derived *D*, with a CV of 2.5 % comparable to the very good repeatability of the monoexponential ADC. Both α and DDC_α_ derived from the stretched exponential model show good CV (3.5 % and 4.3 % respectively), and DDC_K_ derived from the kurtosis model also has a similar CV of 6.1 %. The parameters associated with the fast pseudo-diffusion fraction of the IVIM model, *f* and D*, displayed a significantly higher CV (>30 %) for the full cohort, as did the kurtosis parameter *K*. Within the subgroups for intra- and extracranial tumours, there is no general pattern for comparison of repeatability for the diffusion parameters, with the intracranial cohort displaying lower CV for parameters from the IVIM model, though not for the DDC calculated from either the stretched exponential or kurtosis models.

**Table 3:** Percentage coefficients of variation (%, with 95 % CI in parentheses) for the ROI median of diffusion parameters in each model

	Coefficient of variation (%, with 95 % CI)		
	Intracranial	Extracranial	Full cohort
ADC_100_	4.1 (2.6, 9.0)	2.4 (1.6, 4.9)	3.3 (2.4, 5.1)
*f*	37.6 (23.8, 94.9)	42.3 (27.3, 99.0)	41.0 (29.4, 68.6)
*D*	2.0 (1.3, 4.3)	3.0 (2.0, 6.0)	2.5 (1.8, 4.0)
D*	34.1 (21.6, 84.0)	37.7 (24.5, 85.7)	35.1 (25.4, 57.9)
fD*	26.9 (17.2, 63.6)	46.9 (30.1, 113.0)	38.1 (27.5, 63.4)
DDCα	5.0 (3.2, 11.0)	3.1 (2.0, 6.2)	4.3 (3.2, 6.8)
α	3.0 (1.9, 6.6)	4.1 (2.7, 8.4)	3.5 (2.6, 5.6)
DDC_K_	7.6 (4.9, 16.8)	4.9 (3.2, 10.0)	6.1 (4.4, 9.6)
Kurtosis	38.7 (24.4, 98.3)	59.3 (37.5, 157.6)	52.7 (37.5, 91.7)

ADC_100_ reproduced by permission from ref. [16]

The calculated correlation coefficients between parameters in the different DWI models are given in Table 4 and show high correlations (>0.9) between ADC_100_, IVIM *D*, DDC_α_, and DDC_K_. The stretching exponent α showed moderate negative (<−0.6) correlation with *K*, *f*, and D*. Within models, IVIM showed a moderate correlation between *f* and D* (0.652).

**Table 4:** Pearson correlation coefficient between DWI parameters

	ADC_100_	α	DDC_α_	IVIM *D*	IVIM *f*	IVIM D*	DDC_K_	*K*
ADC_100_	–	0.238	**0.985**	**0.999**	0.166	0.183	**0.910**	*−0.686*
α	0.238	–	0.083	0.238	*−0.805*	*−0.794*	−0.149	*−0.712*
DDC_α_	**0.985**	0.083	–	**0.985**	0.302	0.308	**0.958**	*−0.608*
IVIM *D*	**0.999**	0.238	**0.985**	–	0.151	0.194	**0.906**	*−0.692*
IVIM *f*	0.166	*−0.805*	0.302	0.151	–	*0.652*	0.530	0.391
IVIM D*	0.183	*−0.794*	0.308	0.194	*0.652*	–	0.483	0.270
DDC_K_	**0.910**	−0.149	**0.958**	**0.906**	0.530	0.483	–	−0.439
*K*	*−0.686*	*−0.712*	*−0.608*	*−0.692*	0.391	0.270	−0.439	–

Bold indicates |ρ| > 0.9, italic indicates |ρ| > 0.6

**Fig. 1:**
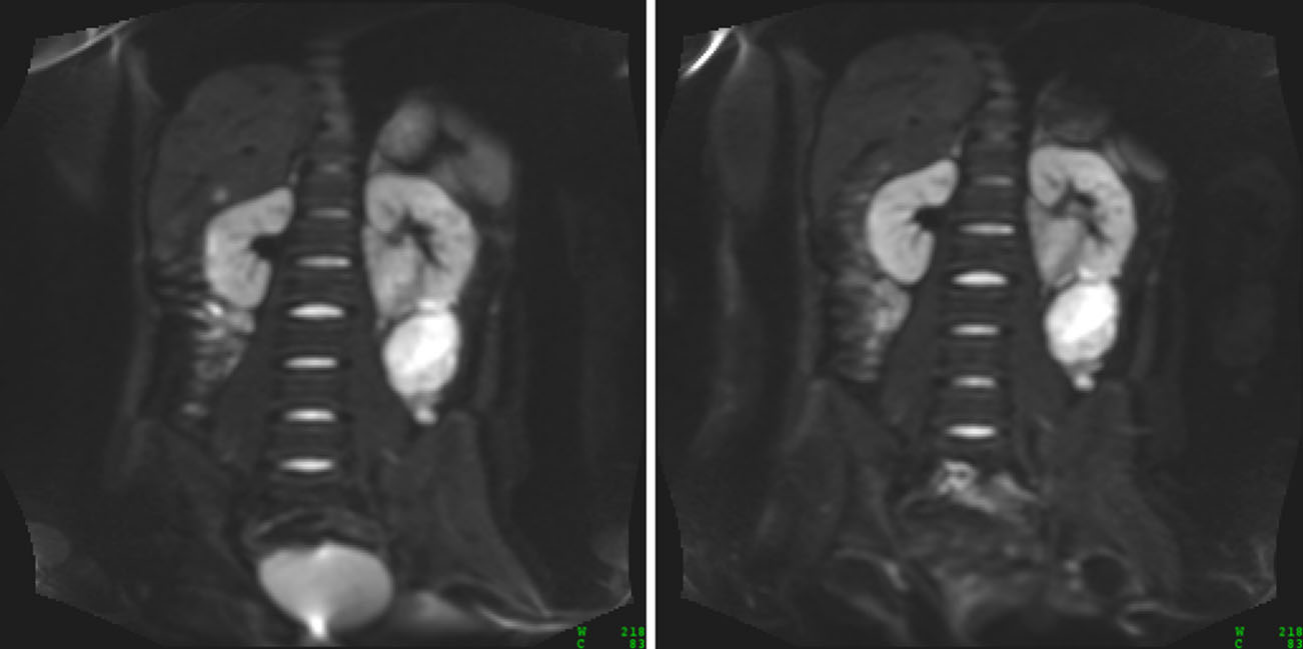
Representative images (*b* = 100 mm^−2^ s) from repeated DWI scans (*left* day 0, *right* day 1) of a patient with rhabdomyosarcoma, showing good visual agreement of patient/tumour positioning

**Fig. 2:**
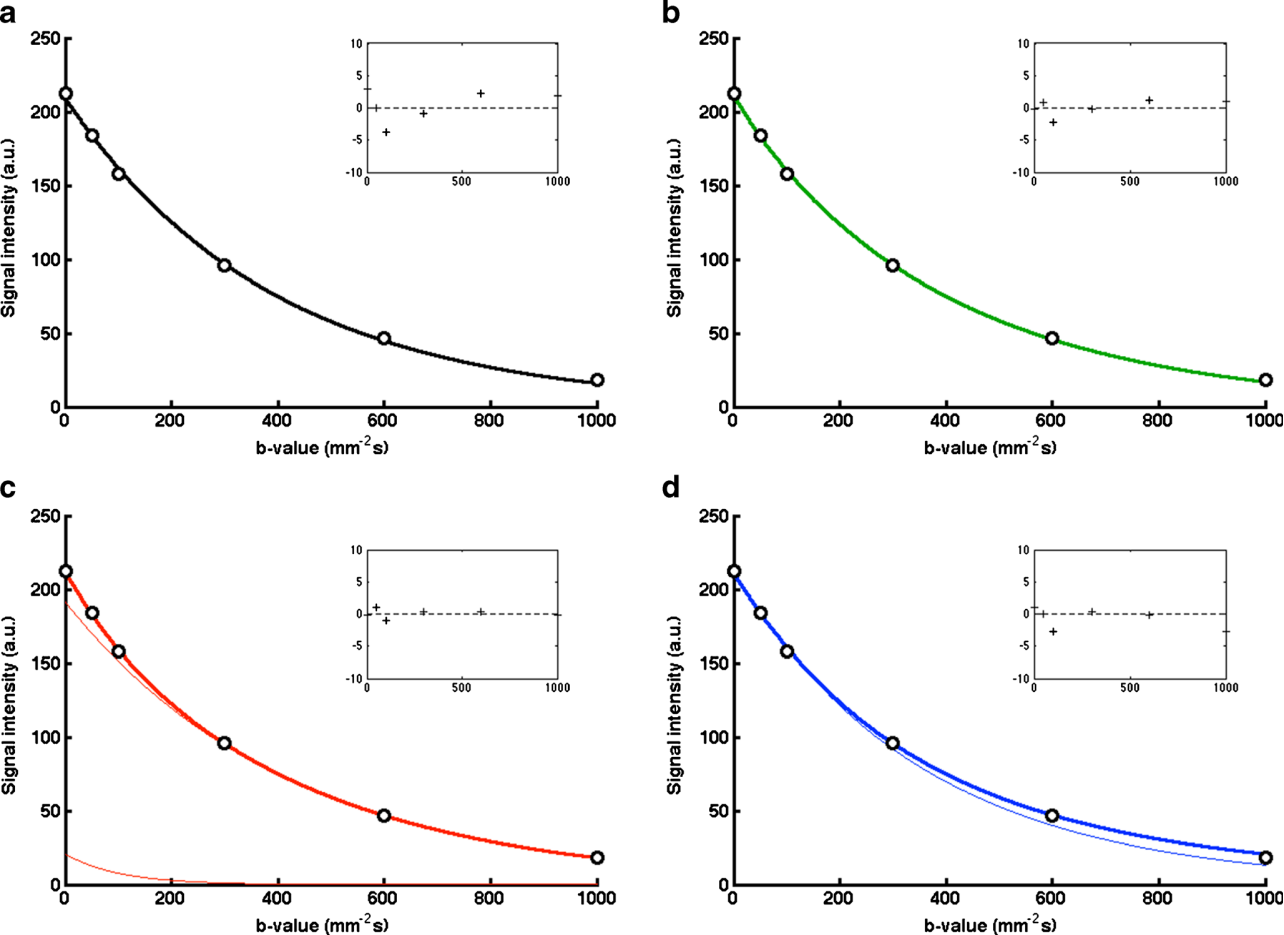
Example plots of diffusion models of data from a single tumour voxel from patient in Fig. 1, using **a** monoexponential, **b** stretched exponential, **c** IVIM, and **d** kurtosis models. *Narrow lines* show **c** the slow and fast components of the model, and **d** the first term in the kurtosis model (DDC_K_). Residuals for the fitting are shown *inset* (units as main axes)

Images at *b* = 100 mm^−2^ s for matched slices on repeated scans in an example patient (a relapsed rhabdomyosarcoma) are given in Fig. 1; the images show an excellent visual agreement. Representative diffusion data for a single voxel within the same tumour displayed in Fig. 1 are shown in Fig. 2; displaying the calculated diffusion models gives a visual indication of where the models differ, including the residuals of the fitting, and how individual model parameters are sensitive to specific regions of the diffusion decay curve.

**Fig. 3:**
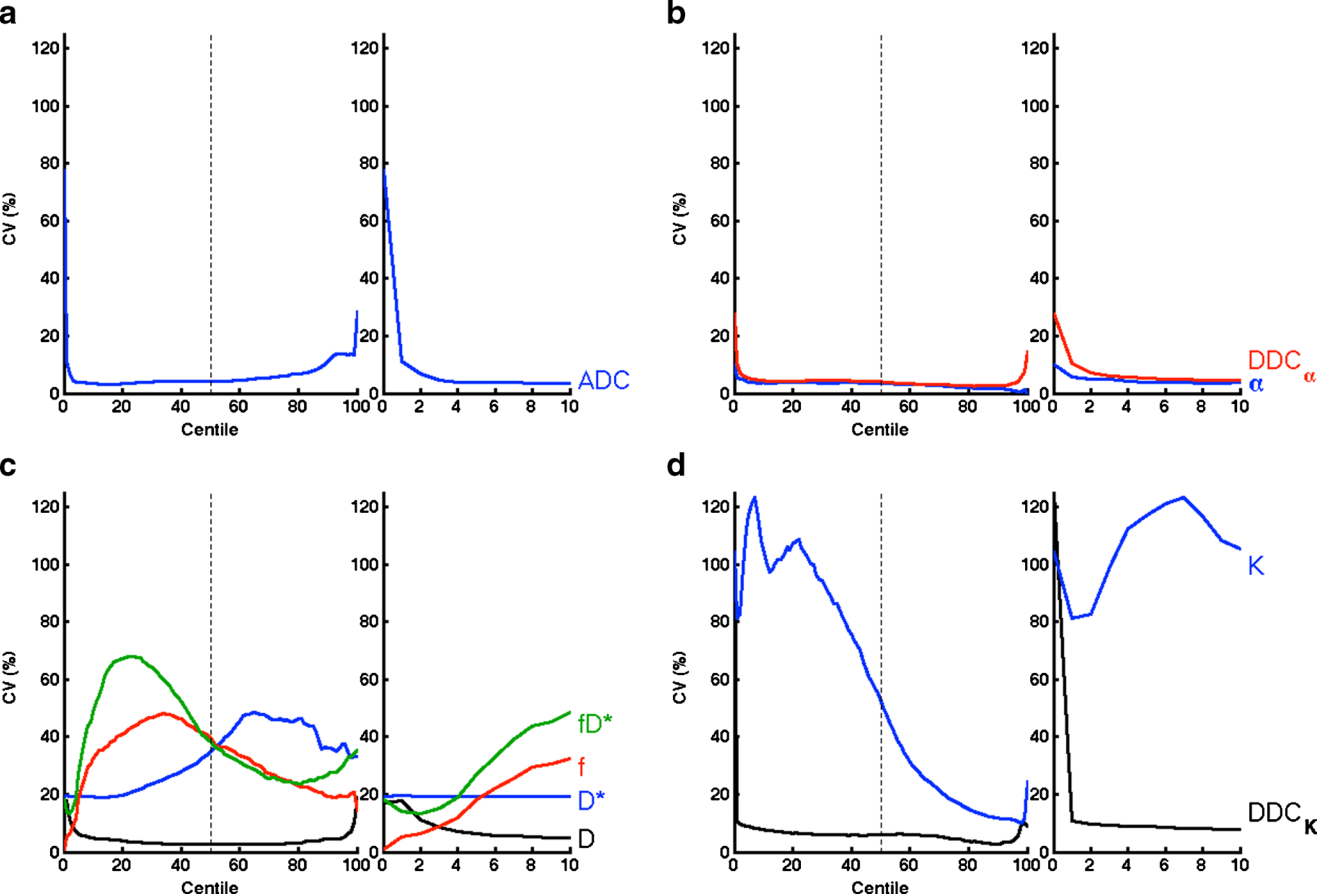
Plots of CV at each centile of the histograms for each parameter in the **a** monoexponential, **b** stretched exponential, **c** IVIM, and **d** kurtosis diffusion models. The diffusion-related parameters exhibit low CV as low as 5th percentile (*expanded sections*), but large variability is observed for perfusion- and kurtosis-related parameters

The CV of the parameters from the different models at each percentile are shown in Fig. 3, showing that the diffusion parameters (ADC, *D*, DDC_α_, and DDC_K_) generally display a characteristic ‘bathtub’ profile, where the smallest and largest percentiles have CVs that are much larger, passing 50 % and even approaching 100 %, either side of a substantially flat region that includes the median. Other model parameters, with the exception of α, display variation from this shape in a non-intuitive way, as well as having higher CV.

## Discussion

The ability to effectively treat cancer is assisted at an individual level by the ability to detect changes in response biomarkers following treatment. Functional imaging techniques have the potential to supply useful imaging biomarkers that provide insight into the mechanism and efficacy of cancer treatment, prediction of patients more likely to respond to a given therapy, as well as providing more physiological sensitivity for earlier assessment of treatment response within and between tumours.

The use of multiple-*b*-value DWI will substantially lengthen an imaging study; this is of greater concern when scanning children, who are prone to greater movement and may find the scan more distressing. Measurements using multiple *b* values, however, allow more complex diffusion models to be applied, which provide additional information reflecting the tumour microenvironment, heterogeneity, and any differential progression or response.

In this study a six-*b*-value DWI protocol, lasting 6 min, was performed in children aged 6 to 15 years with confirmed solid tumours on consecutive days without treatment intervention, in order to assess diffusion parameter repeatability. All patients were able to co-operate throughout the entire imaging protocol, and the functional imaging data obtained was of sufficient quality to allow voxel-wise generation of parametric maps; the repeatability of derived diffusion parameters such as ADC was found to be comparable to that achieved in adult cohorts [12, 16], indicating that multiple-*b*-value DWI is both feasible and repeatable in this cohort.

Limitations of this study include combining both cranial and extracranial tumours in the same evaluation, which may preclude interpretation of DWI parameter values but does not prevent assessment of repeatability behaviour. This study required children to have an additional MRI scan for research purposes only, which resulted in additional ethical and logistical challenges. Nonetheless, repeatability studies typically do not require high numbers to be statistically robust. The study was also limited by exclusion of younger patients, as this would have required a second anaesthesia solely for the research study.

The diffusion models examined in this study attempt to describe any observed deviations from monoexponential decay with *b* value. Estimates of parameters such as fD* and *K* are more heavily influenced by a limited subset of the *b* values (fD* is derived from small *b* values, whereas *K* is not affected by low *b* values), and as observed in these results may be expected to be subject to greater variations (CV > 20 %, and up to 50 % for median) than parameters influenced by a wider range of *b* values, such as ADC_100_. The exponent of the monoexponential decay coefficient, α, is influenced by all *b* values and has a CV similar to that of the ADC and DDC_α_. There remains much interest in the optimisation of *b* values for DWI [23–25], and while the quality of the diffusion fitting may depend on the choice of *b* values, it will also depend on the nature of the target lesion/tissue and the diffusion model choice. For studies that intend to model kurtosis, for example, it may be desirable to replace the lower *b* value acquisitions with some at greater than 1000 mm^−2^ s [26], and IVIM may benefit from added smaller non-zero *b* values that better capture the rapidly changing signal in the pseudo-diffusion sensitive region [27]. The *b* values in this study were chosen to capture a sufficient range of *b* values to apply multiple model fitting, while remaining within a clinically suitable time frame of 6 min, and the lack of *b* values less than 50 mm^−2^ s and greater than 1000 mm^−2^ s may be considered a limitation of the study. Accepting these *b* values as a compromise between coverage and available time, the comparison of diffusion models shows that (excepting the stretched-exponential model) the reproducibility of non-Gaussian diffusion parameters may be limiting their utility, and so any discussion of *b* value number and values must be within that context; optimisation may act to ameliorate this problem.

Examination of the parameter histograms shows an excellent CV for diffusion parameters that are influenced by all *b* values, such as ADC, (IVIM) *D*, DDC_α_, and DDC_K_. Where diffusion coefficients inform on tissue cellularity and extracellular space tortuosity, there is interest in moving from the simple median (or mean) values and evaluating the lowest parameter values that may reflect regions within the tumour that are the most cellular [17]. From Fig. 3, it is remarkable how far the low CV region extends throughout the ROI, and indicates that centiles such as 25th, 10th, and 5th can be used as reliably as the median. The CVs at the very lowest centiles of the histograms, however, shows a high variation that will include sensitivity to data quality, data support, consistency of ROI, size of ROI, and tissue motion. For this reason, properties of these parameter histograms such as the minimum value or below the 5th centile must be considered unreliable, and inferences from such should be avoided. Similarly, the higher CV observed for parameters *f*, D*, and *K* across the whole histogram suggests that interpretation of changes in these parameters as representing changes in underlying physiology is problematic, and should be approached with caution. Higher centiles also suffer the same increased variability and are more likely to reflect outliers from fitting. The perfusion-related parameters in the IVIM model, *f* and D*, and the kurtosis parameter *K* are influenced by smaller subsets of the data (at lower and higher *b* value, respectively) and thus show much greater variability across the histogram. Parameters that have limits imposed during fitting, such as *f* and α, will have artificially reduced CV near the limits, which is a reflection on the characteristics of the data when using that model rather than the utility of the ROI maximum as a functional imaging biomarker. From these data, the stretched exponential model appears to offer a robust and reliable diffusion model that goes beyond that of the monoexponential model, and does not suffer the weaknesses (covariance, high variance, instability across parameter histogram) of IVIM and kurtosis parameters; these observations for different centiles of histograms and diffusion models are not expected to be specific to the paediatric cohort.

High correlation coefficients between ADC_100_, *D*, DDC_α_, and DDC_K_, (correlation coefficients above 0.9 in each case) indicate that they are all reporting on the same phenomenon, taken to be the Brownian diffusion of the observed spins. Within different DWI models, the *f* and D* parameters from the IVIM model have a correlation of 0.65, which although not large indicates that they have an appreciable covariance and are difficult to confidently report (from this acquisition scheme). The very low correlation between α and DDC_α_ in the stretched exponential model (0.083) suggests that these parameters are unique and identifiable parameters [28], associated with independent tissue properties, and thus provide more information than the simple ADC model [29]. The same is true of the pseudo-diffusion-related parameters in the IVIM model, and *K* in the kurtosis model, although the higher CVs observed for these may limit their utility. It is interesting to note that α correlates with both the perfusion and kurtosis parameters of the other models, but the latter show only a very low (<0.4) correlation to each other; this further indicates the stretched exponential model as being suitable for capturing deviation from purely Gaussian diffusion processes.

In conclusion, this study builds on using a well-tolerated free-breathing diffusion imaging protocol to derive and examine the parameters derived from non-Gaussian diffusion models, and demonstrates that while it is possible to obtain repeatable functional parameters beyond the monoexponential ADC, there are large variations seen in parameters that are sensitive to a limited range of *b* values that may hinder useful physiological interpretation. Median values for ADC, *D*, DDC_α_, α, and DDC_K_ had good repeatability (less than 10 %) in the ROI histogram as low as the 5th percentile, but showed sharp increases in variance as the extreme values within the ROI were approached, limiting the usefulness of these values as biomarkers. The poor and variable repeatability of perfusion- and kurtosis-related parameters found across the ROI histogram demonstrates that observed changes in a clinical setting should be interpreted with caution; in contrast, both parameters in the stretched exponential model behaved well. In practice, these results demonstrate the importance of repeatability assessments when considering the interpretation of metrics from advanced analysis of DWI data, including histogram analysis and non-Gaussian diffusion models, in clinical trials that contain functional MR imaging.

## Abbreviations

ADC: apparent diffusion coefficient
CV: coefficient of variation
DCE-MRI: dynamic contrast-enhanced magnetic resonance imaging
DDC: distributed diffusion coefficient
DWI: diffusion-weighted magnetic resonance imaging
EPI: echo-planar imaging
GRAPPA: generalised autocalibrating partially parallel acquisition acceleration
IRB: institutional review board
IVIM: intravoxel incoherent motion
MRI: magnetic resonance imaging
ROI: region of interest
SPAIR: spectral adiabatic inversion recovery
